# Concurrent Cardiac Tamponade and Superior Vena Cava Syndrome: A Concerning Situation

**DOI:** 10.7759/cureus.4253

**Published:** 2019-03-14

**Authors:** Samia Asif, Mobasser Mahmood, Rebecca R Pauly

**Affiliations:** 1 Internal Medicine, University of Missouri Kansas City (UMKC), Kansas City, USA; 2 Cardiology, Mercy Health – St. Vincent Medical Center, Toledo, USA; 3 Internal Medicine, Virginia Tech Carilion School of Medicine, Roanoke, USA

**Keywords:** cardiac tamponade, svc syndrome, diffuse large b-cell non-hodgkin lymphoma

## Abstract

Diffuse large B-cell lymphoma (DLBCL) is the most commonly diagnosed lymphoma; as per the Surveillance, Epidemiology, and End Results (SEER) database 2006-2015, incidence of DLBCL is 7.0/100,000 per year. Superior vena cava (SVC) syndrome and cardiac tamponade are life-threatening oncological emergencies with an overlap in clinical manifestations. While SVC syndrome may commonly be seen with mediastinal masses, literature search shows only one prior case of cardiac tamponade resulting from DLBCL. Here, we present a case of a patient with a concurrent diagnosis of DLBCL and non-small cell carcinoma of the lung (NSCLC), presenting with respiratory symptoms initially but subsequently worsening with hemodynamic compromise. He was found to have cardiac tamponade secondary to DLBCL and was treated appropriately for it but failed to improve clinically due to co-existing SVC syndrome that was not treated. The patient expired in the intensive care unit (ICU) within 24 hours of acute clinical deterioration. This case highlights that in absence of a clinical suspicion for both conditions, identification of one can lead to an overlooked diagnosis of the other. When associated with hemodynamic instability, urgent intervention is mandatory and failure to recognize and treat either of the two may result in grave outcome. This case attempts to alert medical personnel regarding two major oncological emergencies where an accurate diagnosis and urgent intervention can prevent mortality and morbidity.

## Introduction

Currently, lymphomas are the fifth most common systemic malignancies [1]. Diffuse large B-cell lymphoma (DLBCL) is the most common subtype of lymphoma, followed by follicular and Hodgkin’s lymphoma [1]. Primary mediastinal large B-cell lymphoma (PMBCL) is a rare form of non-Hodgkin’s lymphoma (NHL), previously considered a variant of DLBCL but now recognized by the World Health Organization as a distinct entity. PMBCL presents as a large mediastinal mass with infiltration of adjacent structures such as the lung, pleura, and pericardium [2]. While pericardial effusions in setting of underlying malignancy are frequently seen, cardiac tamponade secondary to neoplastic etiology is uncommon [3]. Superior vena cava (SVC) syndrome has been reported with primary cardiac lymphomas and PMBCL. Both SVC syndrome and cardiac tamponade are oncological emergencies. Here we present a unique case of a 71-year-old male with a recent diagnosis of non-small cell carcinoma of the lung (NSCLC) with lymphadenopathy determined to be DLBCL. This is an uncommon case where not only were two primary tumors detected simultaneously, but also the patient deteriorated clinically and was noted to have concurrent SVC syndrome and cardiac tamponade. While SVC syndrome is commonly seen with tumors presenting with mediastinal masses, literature search shows only one prior case of cardiac tamponade resulting from DLBCL [4].

## Case presentation

A 71-year-old Caucasian gentleman with a past medical history of chronic obstructive pulmonary disease (COPD) was admitted with shortness of air and productive cough. A CT angiogram of the chest was obtained due to concerns for pulmonary embolism and showed spiculated nodules in bilateral lower lobes; right nodule measured 1.1 cm x 0.9 cm while the left measured 1.5 cm x 1.2 cm. Also noted was right hilar lymphadenopathy with the largest lymph node measuring 2.9 cm x 2.1 cm; no mediastinal or axillary lymphadenopathy was noted. Emphysematous changes of the lungs were also seen. A biopsy of the left lung nodule and a right lymph node fine needle aspiration (FNA) were performed. Pathology results revealed the left lung nodule to be poorly to moderately differentiated nonkeratinizing squamous cell carcinoma. Cytology studies on the right lymph node sample showed highly atypical large lymphocytes with 95% monoclonal B-cells on flow cytometry, concerning for large B-cell lymphoma. After stabilization from respiratory standpoint, the patient was discharged home on supplemental oxygen therapy via nasal cannula at three liters/minute (L/min). His case was discussed in a multi-disciplinary tumor board. Cardio-thoracic surgery team felt he was not a candidate for lobectomy and would need radiation therapy; a referral to radiation oncology was made. A lymph node excision biopsy was planned to establish a definite diagnosis of lymphoma. 

**Figure 1:**
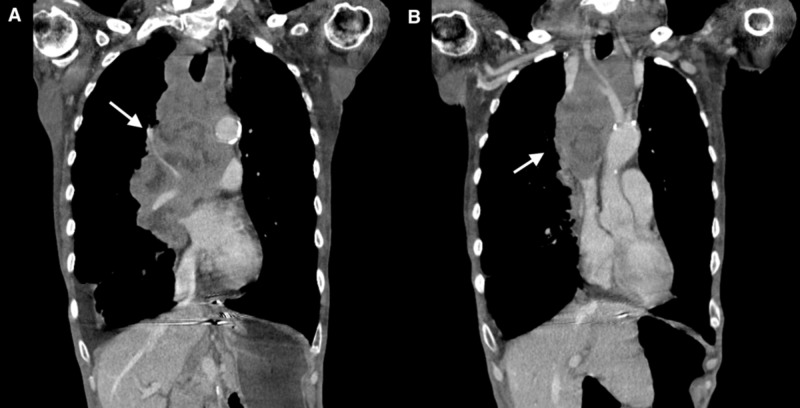
CT chest with contrast revealing large mediastinal mass (A) involving the entire mediastinum and the right hilum and causing (B) narrowing of the superior vena cava.

Approximately eight weeks following his initial admission, the patient presented with worsening dyspnea with productive cough. He was hemodynamically stable with no increase in supplemental oxygen requirements; however, he was noted to have facial swelling, jugular venous distention, diffuse wheezing with bilateral basal crackles and bilateral pitting pedal edema. Treatment was started for acute COPD exacerbation. Intravenous (IV) furosemide was given as well as given peripheral edema along with prednisone 50 mg daily. A CT chest with contrast was performed; this showed a large, centrally necrotic 18.1 cm x 8.7 cm x 9.7 cm bilateral lobulated infiltrating mediastinal mass involving the entire mediastinum and the right hilum, causing mass-effect upon the heart, superior and inferior vena cava and pulmonary arteries and deforming the airway including trachea and right main bronchus. Significant narrowing of the SVC, the right main and right upper lobe pulmonary arteries was seen as well as of the left brachiocephalic vein. Multifocal consolidations were also noted. Bilateral lung nodules were re-visualized but were stable in size in comparison to prior CT scan. Small pericardial effusion was seen (Figure 1).

**Figure 2:**
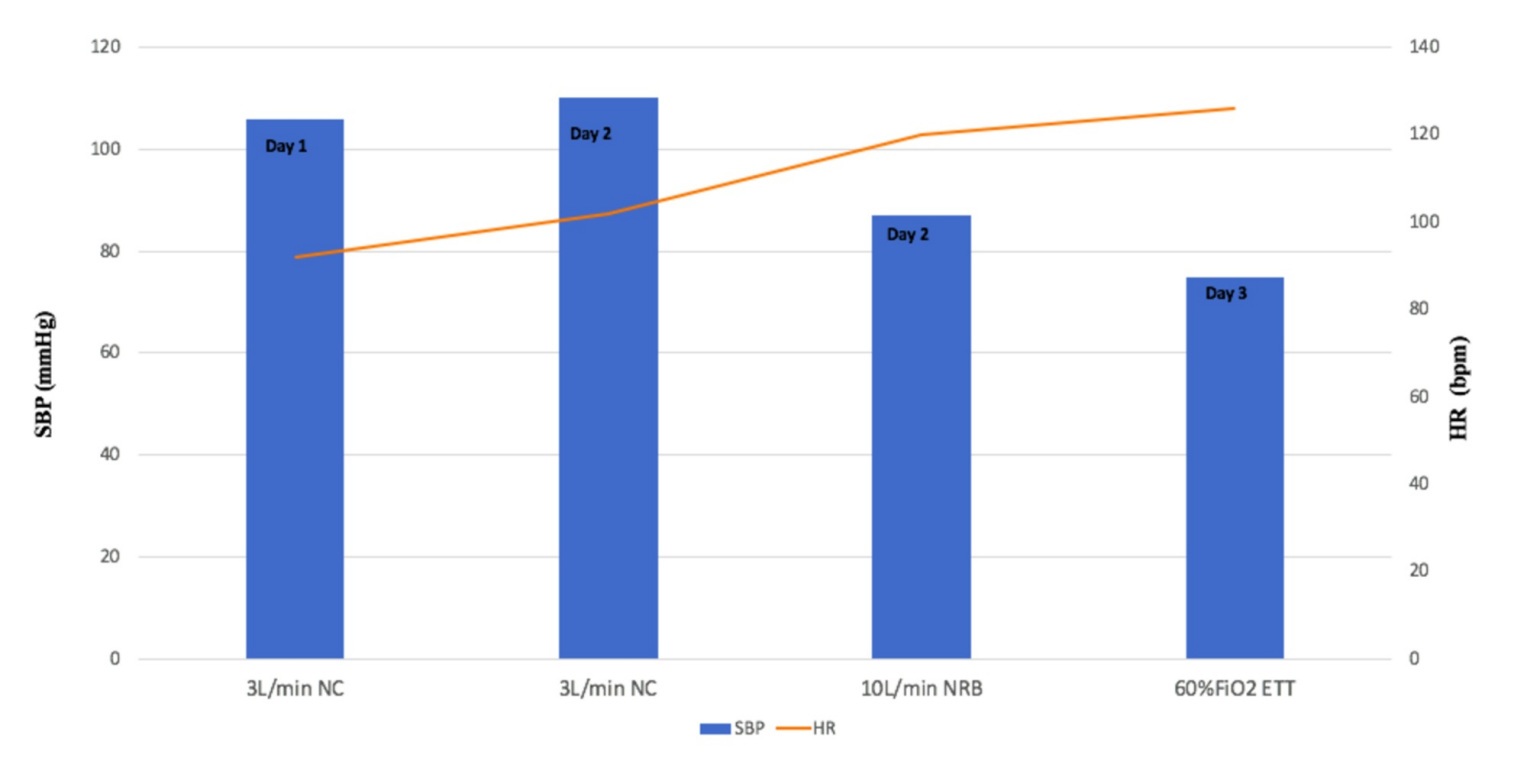
Trend in patient's vitals since admission revealing tachycardia, hypotension, and increased oxygen requirements. SBP, systolic blood pressure (mmHg); HR, heart rate (beats per minute); NC, nasal cannula; NRB, non-rebreather mask; ETT, endotracheal tube; FiO2, fraction of inspired oxygen.

Blood and sputum cultures were obtained; broad spectrum antibiotics including vancomycin, cefepime, and levofloxacin were initiated for pneumonia in setting of recent hospitalization. The mediastinal mass was presumed to be rapidly enlarging lymphoma. Bone marrow and left supra-clavicular lymph node biopsy was obtained by interventional radiology (IR). Over the next 24 hours, he was observed to have increasing tachypnea; oxygen requirement increased from 3 L/min by nasal cannula (NC) to 10 L/min via a non-rebreather (NRB) mask. On examination, the patient was noted to have audible stridor. He acutely became hypotensive; systolic blood pressure (SBP) dropped from 110 mmHg and above up until this point to below 90 mmHg. The deterioration in his hemodynamic and oxygenation status is depicted in Figure 2.

**Figure 3:**
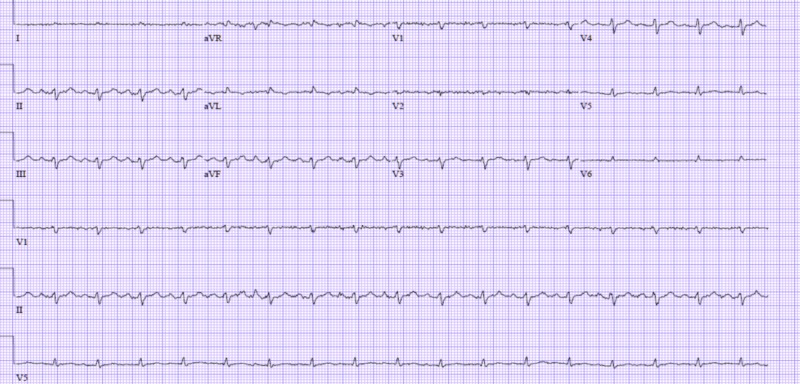
Electrocardiogram revealing low voltage QRS complexes and sinus tachycardia.

He was transferred to the intensive care unit (ICU) and was subsequently intubated. Inotropic support was initiated. An arterial line waveform was suggestive of pulsus paradoxus. Electrocardiogram (EKG) showed sinus tachycardia at a rate of 110 beats per minute (bpm) with low voltage QRS (Figure 3). 

A trans-thoracic echocardiogram (TTE) was immediately performed which showed moderate-sized pericardial effusion with signs concerning for impending cardiac tamponade including right ventricle diastolic collapse. The patient was urgently taken for diagnostic and therapeutic pericardiocentesis; 160 milliliters (mL) of pericardial fluid was drained followed by placement of a pigtail catheter in the pericardial sac. Following the procedure, the patient became increasingly hypotensive and inotropic support was intensified with addition of epinephrine followed by vasopressin to an already maximum rate of norepinephrine. The IV fluids in periodic boluses were given with temporary improvement in blood pressure each time. A repeat TTE showed only mild pericardial effusion. Over the next few hours, the patient developed worsening swelling of his face, upper chest, and upper extremities. He was also noted to have worsening abdominal distention. Eventually, the patient's urine output declined. Pericardial fluid analysis showed bloody appearance with 71% mononuclear cells; malignant lymphoma cells were noted on cytology. His recent lymph node biopsy results were reviewed and a preliminary read was consistent with aggressive DLBCL. Dose adjusted etoposide, prednisone, vincristine, cyclophosphamide, and doxorubicin (DA-EPOCH) were started promptly by the oncology team who felt that the lymphoma was chemo-sensitive and a rapid response to treatment could be anticipated. Prior to chemotherapy initiation, a renal panel was obtained, revealing metabolic acidosis (bicarbonate levels 14.2 mmol/L, pH 7.022 on arterial blood gas), hyperkalemia (potassium 6.0 mEq/L), hyperphosphatemia (phosphorus 6.3 mg/dL), and hypocalcemia (calcium 6.2 mg/dL). Nephrology was consulted for initiation of continuous renal replacement therapy (CRRT) in setting of oliguria and concerns for tumor lysis syndrome (TLS). His family requested for comfort care measures to be pursued at this point; the patient was extubated palliatively and expired within a few minutes. Later, the patient’s respiratory cultures grew *Acinetobacter baumannii* which was only intermediately sensitive to cefepime, meropenem, and levofloxacin (Table 1).

**Table 1:** Antibiotic sensitivity of Acinetobacter baumannii detected from patient's respiratory culture.

Drug	Acinetobacter baumannii
Piperacillin-Tazobactam	Resistant
Ceftriaxone	Resistant
Levofloxacin	Intermediate
Meropenem	Intermediate
Cefepime	Intermediate
Gentamicin	Sensitive
Tobramycin	Sensitive
Trimethoprim-Sulfamethoxazole	Sensitive

## Discussion

Emergencies in oncology can involve multiple organ systems. Examples include acute airway obstruction, febrile neutropenia, malignant spinal cord compression, and metabolic abnormalities such as tumor lysis syndrome.

Pericardial effusions may result from the malignancy itself, either by secondary metastases to the pericardium or by direct invasion of the pericardium. Alternatively, it may be a consequence of radiation therapy or concurrent infection. Lymphoma is reported as underlying etiology for only 8%-21% of malignant pericardial effusions [5]. Pericardial effusions may be asymptomatic and incidentally discovered or can result in significant symptoms such as shortness of air, chest pain, and cough. Physical examination may reveal tachycardia, hypotension, distant heart sounds, peripheral edema, pulsus paradoxus, or fixed jugular venous distention. EKG may show low voltage; sometimes electrical alternans, that is, a variation in the amplitude and axis of QRS-complex with each beat, is seen. TTE is diagnostic; a CT or an MRI can provide additional details such as presence of direct tumor invasion of the pericardium. Pericardial fluid cytology may reveal malignant cells. In patients with acute symptoms and hemodynamic compromise, therapeutic echocardiogram-guided pericardiocentesis provides immediate relief; pericardial drain can subsequently be placed. Surgical intervention or sclerosing agent instillation may be needed; systemic chemotherapy and radiotherapy can also prevent re-accumulation in some cancers [6-7].

Superior vena cava syndrome occurs if there is occlusion or external compression of the SVC resulting in poor venous drainage from the upper extremities, the head, and the neck. Symptoms may include shortness of breath, orthopnea, facial congestion, or a headache exacerbated by stooping. Physical examination may reveal swelling of the face, neck, and upper extremities; dilated veins may be seen on the neck, the chest, and the proximal arms. When SVC syndrome leads to laryngeal edema or increased intracranial pressure, the patient may be noted to have stridor and mental status changes, respectively. The CT chest with IV contrast allows diagnosis. In cases of hemodynamic instability, airway compromise, or concerns for elevated intracranial pressure, urgent treatment is needed. Placement of an endovascular stent in the SVC can relieve symptoms immediately. Radiation therapy while effective takes time to provide symptom relief. Elevating the head end of the bed, providing supplemental oxygen, steroid use, and diuretics can be given as further supportive therapy [8]. SVC syndrome is reported only in 4% patients with NHL with 60% of these cases being PMBCL. Only 21% of cases with DLBCL and lymphoblastic lymphoma are associated with SVC syndrome [9].

Diagnosis of DLBCL requires histopathological evaluation of a sample obtained from the involved tissue; in case of lymphadenopathy, excisional biopsy of an affected lymph node is performed. Patients with localized disease, including stage one (single lymph node region or organ involvement) and limited stage two disease (adjacent lymph nodes or one organ with adjacent lymph nodes involvement), rituximab with cyclophosphamide, doxorubicin, vincristine, and prednisone (R-CHOP) for four cycles with involved site radiation therapy (ISRT) or six cycles without radiation can be used. However, if there is bulky disease with a mass more than 7.5 cm in size, six cycles of R-CHOP with radiation therapy can be used; exact therapy is individualized for each patient. For disseminated disease (stage three and four), after two to four cycles of therapy, re-staging is done to confirm treatment response. If a good therapeutic response is observed, R-CHOP is continued for six cycles and ISRT to bulky (mass size more than 7.5 cm) disease is provided; then observation with periodic labs and imaging is recommended. If only a partial response or progressive disease is seen, then depending on whether a patient is a candidate for autologous hematopoietic stem cell transplantation, decision to proceed with second-line regimens versus enrollment in clinical trials, palliative ISRT or supportive care is made [10].

## Conclusions

Concurrent presence of a malignant pericardial effusion which leads to cardiac tamponade as well as SVC syndrome is an uncommon clinical presentation and one that is particularly concerning. They may have similar clinical presentations; in absence of clinical suspicion for both, identification of one can lead to an overlooked diagnosis of the other. Each of the two may be associated with hemodynamic instability necessitating urgent intervention; failing to intervene may result in grave outcome, such as in our patient where while cardiac tamponade was appropriately managed emergently, SVC syndrome was not. Unfortunately, his clinical status was further compromised by underlying pneumonia with resistant Acinetobacter. Accurate diagnosis and urgent intervention is essential to reduce mortality and morbidity in patients who present with oncological emergencies.
